# Minimally invasive vs. open segmental resection of the splenic flexure for cancer: a nationwide study of the Italian Society of Surgical Oncology-Colorectal Cancer Network (SICO-CNN)

**DOI:** 10.1007/s00464-022-09547-6

**Published:** 2022-09-09

**Authors:** Maurizio Degiuli, Monica Ortenzi, Mariano Tomatis, Lucia Puca, Desiree Cianflocca, Daniela Rega, Annalisa Maroli, Ugo Elmore, Francesca Pecchini, Marco Milone, Roberta La Mendola, Erica Soligo, Simona Deidda, Domenico Spoletini, Diletta Cassini, Alessandra Aprile, Michela Mineccia, Herald Nikaj, Francesco Marchegiani, Fabio Maiello, Cristina Bombardini, Michele Zuolo, Michele Carlucci, Luca Ferraro, Armando Falato, Alberto Biondi, Roberto Persiani, Patrizia Marsanich, Daniele Fusario, Leonardo Solaini, Sara Pollesel, Gianluca Rizzo, Claudio Coco, Alberto Di Leo, Davide Cavaliere, Franco Roviello, Andrea Muratore, Domenico D’Ugo, Francesco Bianco, Paolo Pietro Bianchi, Paola De Nardi, Marco Rigamonti, Gabriele Anania, Claudio Belluco, Roberto Polastri, Salvatore Pucciarelli, Sergio Gentilli, Alessandro Ferrero, Stefano Scabini, Gianandrea Baldazzi, Massimo Carlini, Angelo Restivo, Silvio Testa, Dario Parini, Giovanni Domenico De Palma, Micaela Piccoli, Riccardo Rosati, Antonino Spinelli, Paolo Delrio, Felice Borghi, Marco Guerrieri, Rossella Reddavid

**Affiliations:** 1 University of Turin, Department of Oncology, San Luigi University Hospital, Div of Surgical Oncology, Orbassano, Turin, Italy; 2 Clinica Chirurgica Universita’ Politecnica delle Marche, Ospedali Riuniti, Ancona, Italy; 3 BSIT, Department of Oncology, University of Turin, Orbassano, Turin, Italy; 4 Department of Surgery, S. Croce e Carle Hospital, Cuneo, Italy; 5 Department of General and Emergency Surgery, Azienda Ospedaliero Universitaria, Città della Salute e della Scienza, Turin, Italy; 6 Colorectal Surgical Oncology, Abdominal Oncology Department, Fondazione Giovanni Pascale IRCCS, Naples, Italy; 7 Colon and Rectal Surgery Division, Humanitas Clinical and Research Center, Via Alessandro Manzoni, 56, Rozzano, 20089 Milan, Italy; 8 Division of Gastrointestinal Surgery, Vita Salute University, San Raffaele Hospital, 20132 Milan, Italy; 9 Unita’ Operativa di chirurgia generale, d’urgenza e nuove tecnologie, OCSAE, Azienda Ospedaliero Universitaria di Modena, Modena, Italy; 10 Department of Clinical Medicine and Surgery, Department of Gastroenterology, Endocrinology and Endoscopic Surgery, University of Naples “Federico II”, Naples, Italy; 11 General Surgery Unit, Santa Maria della Misericordia Hospital, Rovigo, Italy; 12 S.C. Chirurgia Generale, Ospedale S. Andrea, Vercelli, Italy; 13 Chirurgia Coloproctologica-AOU Cagliari, Dipartimento di Scienze Chirurgiche, Università di Cagliari, Cagliari, Italy; 14 UOC Chirurgia Generale, Ospedale S. Eugenio, Piazzale dell’Umanesimo, 10, 00144 Rome, Italy; 15 Unità Operativa Complessa di Chirurgia Generale, P.O. SSG, ASST NORD MILANO, Milan, Italy; 16 Surgical Oncology Surgery, IRCCS Policlinico San Martino, Genoa, Italy; 17 Department of General and Oncological Surgery, ”Umberto I” Mauriziano Hospital, Turin, Italy; 18 SCDU Clinica Chirurgica, General Surgery Department, AOU “Maggiore Della Carità” Hospital, Novara, Italy; 19 Department of Surgical, Oncological, and Gastroenterological Sciences, University of Padua, Padua, Italy; 20 Department of Surgery, General Surgery Unit, Hospital of Biella, Biella, Italy; 21 Department of Surgical Morphology and Experimental Medicine, AOU Ferrara, Ferrara, Italy; 22 General Surgery Division, “Valli del Noce” Hospital, Cles, Provincial Agency for Health Services (APSS), Trento, Italy; 23 Gastrointestinal Surgery, San Raffaele Hospital, 20132 Milan, Italy; 24 Division of General and Robotic Surgery, Dipartimento di Scienze della Salute, Università di Milano, 20142 Milan, Italy; 25 General Surgery Unit, San Leonardo Hospital, ASL-NA3sud, Castellammare di Stabbia, Naples, Italy; 26 Fondazione Policlinico Gemelli, IRCCS, AREA di Chirurgia Addominale, Rome, Italy; 27 Surgical Department, Edoardo Agnelli Hospital, Pinerolo, Italy; 28 UOC General and Oncological Surgery, University of Siena, Siena, Italy; 29 General and Oncologic Surgery, Morgagni-Pierantoni Hospital, Ausl Romagna, Forlì, Italy; 30 Fondazione Policlinico Universitario A. Gemelli, IRCCS, Chirurgia Generale Presidio Columbus, Rome, Italy; 31 UOC di Chirurgia, Ospedale “San Camillo”, Trento, Italy; 32 Department of Surgery, Misericordia Hospital, Grosseto, Italy; 33 Department of Surgical Oncology, CRO Aviano, National Cancer Institute, IRCCS, Aviano, Italy; 34 UOC Chirurgia Generale, Ospedale S. Eugenio, Piazzale dell’umanesimo, 10, 00144 Rome, Italy; 35 IRCCS Humanitas Research Hospital, via Manzoni 56, 20089 Rozzano, Milan, Italy; 36 Department of Biomedical Sciences, Humanitas University, Via Rita Levi Montalcini 4, 20072 Pieve Emanuele, Milan, Italy; 37 Oncological Surgery, Candiolo Cancer Institute-FPO-IRCCS, Candiolo, 10060 Torino, Italy; 38 Department of Oncology, Head Surgical Oncology and Digestive Surgery, University of Torino, San Luigi University Hospital, Regione Gonzole 10 Orbassano, 10043 Turin, Italy

**Keywords:** Splenic flexure cancer, Minimally invasive surgery, Open surgery, Colon cancer, Segmental resection, Laparoscopic resection

## Abstract

**Background:**

Evidence on the efficacy of minimally invasive (MI) segmental resection of splenic flexure cancer (SFC) is not available, mostly due to the rarity of this tumor. This study aimed to determine the survival outcomes of MI and open treatment, and to investigate whether MI is noninferior to open procedure regarding short-term outcomes.

**Methods:**

This nationwide retrospective cohort study included all consecutive SFC segmental resections performed in 30 referral centers between 2006 and 2016. The primary endpoint assessing efficacy was the overall survival (OS). The secondary endpoints included cancer-specific mortality (CSM), recurrence rate (RR), short-term clinical outcomes (a composite of Clavien-Dindo > 2 complications and 30-day mortality), and pathological outcomes (a composite of lymph nodes removed ≧12, and proximal and distal free resection margins length ≧ 5 cm). For these composites, a 6% noninferiority margin was chosen based on clinical relevance estimate.

**Results:**

A total of 606 patients underwent either an open (208, 34.3%) or a MI (398, 65.7%) SFC segmental resection. At univariable analysis, OS and CSM were improved in the MI group (log-rank test *p* = 0.004 and Gray’s tests *p* = 0.004, respectively), while recurrences were comparable (Gray’s tests *p* = 0.434). Cox multivariable analysis did not support that OS and CSM were better in the MI group (*p* = 0.109 and *p* = 0.163, respectively). Successful pathological outcome, observed in 53.2% of open and 58.3% of MI resections, supported noninferiority (difference 5.1%; 1-sided 95%CI − 4.7% to ∞). Successful short-term clinical outcome was documented in 93.3% of Open and 93.0% of MI procedures, and supported noninferiority as well (difference − 0.3%; 1-sided 95%CI − 5.0% to ∞).

**Conclusions:**

Among patients with SFC, the minimally invasive approach met the criterion for noninferiority for postoperative complications and pathological outcomes, and was found to provide results of OS, CSM, and RR comparable to those of open resection.

**Supplementary Information:**

The online version contains supplementary material available at 10.1007/s00464-022-09547-6.

Despite recent improvements in prevention, early diagnosis, and treatment, colon cancer (CC) is the second leading cause of cancer-related death [1]. To date, surgery is the only treatment for locally advanced disease, being well defined for most tumor locations. Anatomical standard resections are worldwide accepted for right- and left-sided cancers, while tumors of the transverse colon (TC) and of the splenic flexure (SF) are actually treated either with extended or segmental colectomies [2].

Both these cancers show a lower incidence as compared with other locations and are characterized by a poor prognosis [3–6]; mostly due to a variable lymphatic drainage and a late diagnosis. Hence, many authors have suggested extensive resections with the aim to improve oncological outcomes [7–10]. However, the optimal surgical treatment concerning postoperative and oncological outcomes is still under debate [11–13].

Moreover, several studies have investigated not only the extension of colonic resection and lymphadenectomy, but also the type of approach, comparing open (O) versus minimally invasive (MI) surgery [14–16].

Effectively, MI surgery has spread substantially over the past decade, and access to laparoscopic equipment is now nearly universal [17]. Still, wide variations in the adoption of laparoscopic approach across different hospital types and geographic regions exist, but referral centers and teaching hospitals all over the world have certainly acquired the skill to face all CC surgery without the need to open the abdomen.

However, despite this spread, to date, no trial has documented a real survival advantage of MI vs O surgery for any location of CC, except from a single-center, low-volume trial which harbored several limitations [18].

Recently, our nationwide retrospective study including 1304 patients with SF cancers (SFCs) submitted to surgery assessed the safety and efficacy of segmental as compared to extended resection [13]. In this study, segmental resection was proved to be an adequate option in case of colonic cancer at this site. This procedure was performed in 791 patients, and an MI approach was adopted in about 60% of them.

This subset of patients’ population has been used here to analyze the early and oncological outcomes of MI as compared to open approach.

This study aimed to investigate whether the MI approach is noninferior to open resection, as determined by assessment of postoperative complications and pathological evaluation of the resected specimen and if survival and recurrence are at least comparable to those reported after open procedures.

## Materials and methods

### Patients

The details of the design and methods of our previous SICO-CCN retrospective study have been reported formerly [13]. All consecutive patients submitted to *segmental resection* between January 2006 and December 2016 in 30 referral Italian centers were included in this new study. Patients younger than 18 years, with a primary diagnosis of inflammatory bowel or diverticular disease, and with benign or metastatic tumors were excluded. Demographic, operative, pathological characteristics and oncological outcomes were collected in a dedicated database.

The study was approved by the institutional review boards of each participating center. Written informed consent was obtained from all participants.

Data were collected from each participating center’s data manager (Tab 1 suppl). Data managers were not blinded to the intervention.

With the aim to render more homogeneous the two arms of the study, patients with pT4 and ASA IV and those submitted to emergency surgery were excluded from the analyses.

### Preoperative assessment

Patients underwent the same preoperative work-up including clinical examination, total colonoscopy for the identification, biopsy and tattooing of the malignancy, determination of serum levels of carcinoembryonic antigen (CEA) and CT scan to stage locoregional tumor extension and distant metastases according to ESMO guidelines [19].

Patients with bowel obstruction, perforation or massive hemorrhage were managed in emergency setting and submitted to preoperative endoscopy only in case of indication to trans-tumoral stenting as a bridge to surgery.

### Interventions

Resection of the SF was defined as the resection of the colon between the left branch of the middle colic artery (LBMCA) and the left colic artery (LCA); these vessels were ligated centrally, at their origin from the main trunk of the middle colic artery (MCA) and from the inferior mesenteric artery (IMA), respectively; the anastomosis was performed between the middle transverse and the middle descending colon [20].

As this study was retrospective, MI and open approaches were used according to the individual preference of participating surgeon.

#### Laparoscopic approach

After a 12 mmHg pneumoperitoneum was created either using Hasson open entry or Veress needle laparoscopy technique, the procedure started with exploring the abdomen, identifying critical structures, and ligating the main vascular pedicles (i.e., the LBMCA and the LCA). After proximal and distal colonic transection with at least 5 cm free margin length at both sides, the anastomosis was performed either manually or mechanically and intra- or extra-corporeally depending on surgeon confidence [21–23].

#### Robotic approach

The robotic SF resection was similar to laparoscopic procedure, with the exception of ports placement which is standardized with their location along an oblique line connecting the anterior superior iliac spine and the left costal margin [24].

#### Open approach

Open SF resections were performed following the same principles of dissection, vascular control, and oncological colonic resection described for minimally invasive approach. Midline and left Kocher incisions were mostly adopted.

### Pathology

Pathological staging was performed according to the American Joint Committee on Cancer (AJCC) staging system. Every case was re-classified according to the TNM 8^th^ edition [25].

Both distal and proximal free margin length were assessed in the fresh and unstretched specimen. Residual tumor and lymphatic/vascular invasion were evaluated as reported previously [13]. The number of lymph nodes retrieved was assessed in the prepared surgical specimen.

### Endpoints of the study

The primary endpoint was the overall survival (OS). Secondary endpoints included cancer-specific mortality and recurrence rate as well as early outcomes, namely short-term clinical outcomes (a composite of the rate of Clavien–Dindo postoperative complications > grade 2, and of postoperative mortality at 30 days from operation) and pathological outcomes (a composite of a number of lymph nodes removed ≧12 and of proximal and distal free resection margins length ≧ 5 cm).

### Statistical analysis

A first analysis was performed comparing variables between groups with open and MI approach. Demographic and clinical characteristics were presented as distribution frequencies. Quantitative and qualitative variables were described using medians/quantiles and frequencies/percentages, respectively. Differences in continuous variables were tested using the Kruskal–Wallis test, because all were not normally distributed (normality was checked with Shapiro–Wilk test). For categorical variables, χ^2^ test or Fisher’s exact test were used as appropriate.

For short-term clinical and pathological outcomes, a noninferiority analysis was performed. The short-term clinical outcome assessing efficacy was a composite of postoperative complications grade < 3 according to Clavien–Dindo classification and 30-day postoperative survival. The pathologic outcome assessing efficacy was a composite of proximal and distal free resection margin length of at least 5 cm and at least 12 lymph nodes retrieved. For both these measures, a 6% noninferiority margin was chosen according to clinical relevance estimation.

Overall survival (OS) was calculated using the Kaplan–Meier method. Competing risks analysis was performed to assess cancer-specific mortality (CSM) and time to recurrence. For OS and CSM comparisons in ten variables chosen for their clinical relevance (age, sex, intervention, splenic resection, ASA, ECOG, ACCI, TNM, total anesthetic time, and volume) were performed with the Cox proportional hazards model; the choice considered the risk of omitting relevant variables (for the omitted variable bias) balanced by the actual clinical relevance of the set of variables selected for the univariable analysis. The same set of ten variables was included in a saturated model. A backward stepwise procedure was used to identify the most relevant covariates, excluding the records with missing information in the variables.

Multivariable OS analysis was performed in the 577 cases with information available on all ten covariates; ACCI, ASA, and total anesthetic time were excluded by backward stepwise procedure.

Cancer-specific mortality analysis was performed in the 563 cases with information available on the outcome and all the ten covariates; ECOG, ASA, and total anesthetic time were excluded by backward stepwise procedure.

For recurrence analysis, no multivariable analysis was performed, because at univariable analysis, no correlation with intervention was found.

All statistical analyses were performed using R version 4.0.5 (© The R Foundation); statistical significance was set at *p* < 0.05.

This is an ancillary study, which involves the derivation of data for purposes that are separate from the main trial we have published previously (*). Actually, a pre-specified study protocol concerning this new study is not available.

## Results

### Patient characteristics and perioperative details

**Fig. 1:**
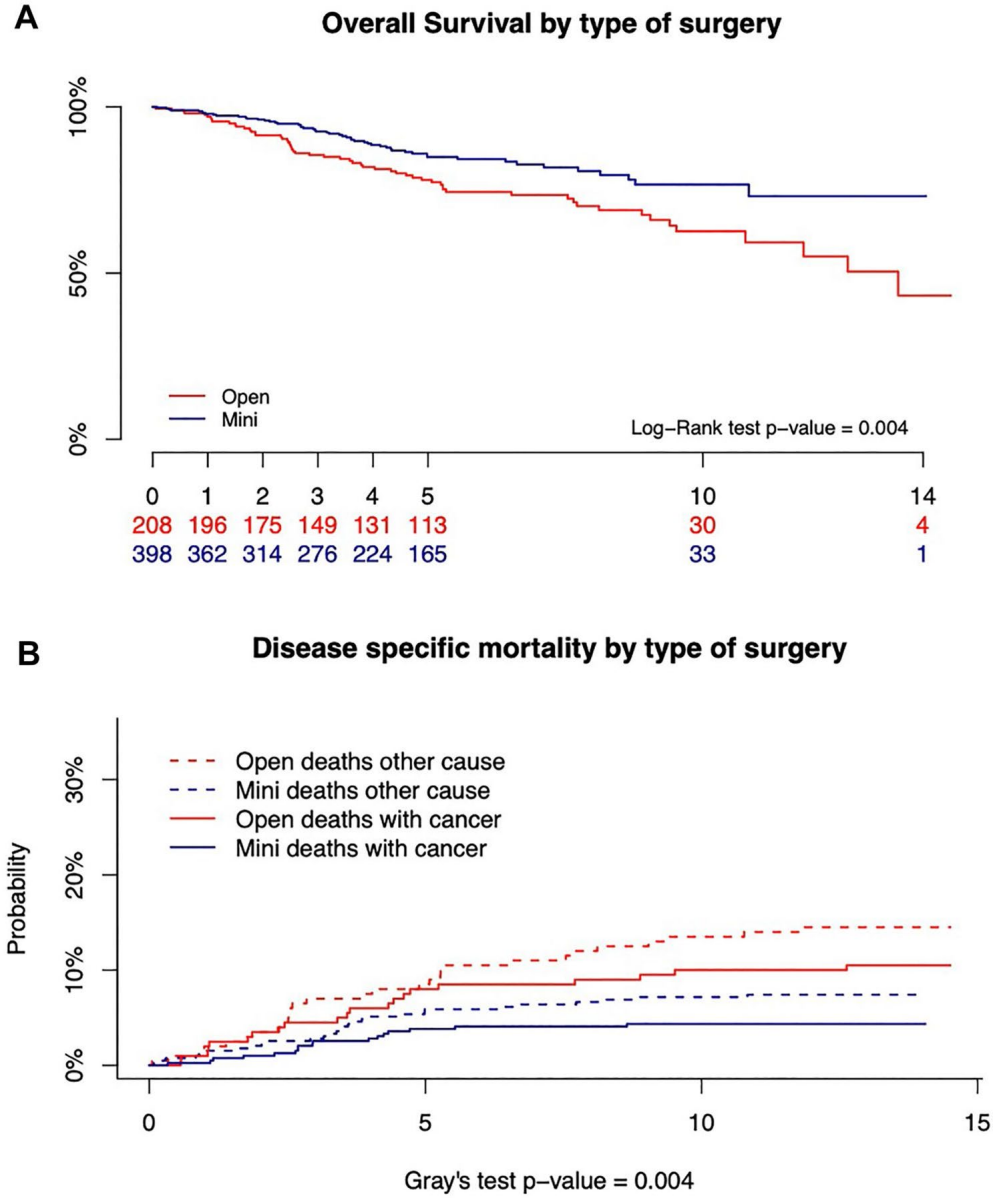
**A** Overall survival by type of surgical approach. The 10-year OS was significantly higher in MI than in O group: 76.6% (95% CI 70.1–83.8%) and 62.6% (95% CI 54.0–72.4%), respectively (log-rank test *p* = 0.006). **B** Mortality for colon cancer by type of surgical approach. The proportion of deaths for colon cancer was significantly lower in MI as compared to O group: 7.9% (95% CI 3.4–12.2%) and 16.4% (95% CI 8.2–23.9%) after 10 years, respectively (Gray’s test *p* = 0.004)

After removal of patients with ASA IV (nr. 12), pT4 (nr. 85) or submitted to emergency surgery (nr. 32), a total of 606 patients who underwent either an open (208, 34.3%, O group) or a MI (398, 65.7%, MI group) splenic flexure segmental resection were included in this study (Fig. 1 suppl). Patients’ baseline characteristics are shown in Table 1.

**Table 1:** Patients’ characteristics

		O	MI	Total	*p* value *
No. cases		208	398	606	
Sex	F	86 (41.3)	180 (45.2)	266 (43.9)	0.408
	M	122 (58.7)	218 (54.8)	340 (56.1)	
Age	Median (IQR)	71.0 (64.0 to 78.0)	69.0 (62.0 to 77.0)	70.0 (63.0 to 77.0)	0.075
	(Missing)	1	1	2	
BMI	Median (IQR)	26.4 (23.4 to 28.5)	25.0 (23.0 to 27.3)	25.3 (23.1 to 27.7)	**0.010**
	(Missing)	87	79	166	
ECOG	0–1	150 (72.1)	285 (71.6)	435 (71.8)	0.871
	2 +	57 (27.4)	103 (25.9)	160 (26.4)	
	(Missing)	1 (0.5)	10 (2.5)	11 (1.8)	
ACCI	2–5	144 (69.2)	287 (72.1)	431 (71.1)	0.487
	6 +	64 (30.8)	110 (27.6)	174 (28.7)	
	(Missing)	0 (0.0)	1 (0.3)	1 (0.2)	
ASA	1–2	109 (52.4)	267 (67.1)	376 (62.0)	**0.001**
	3	93 (44.7)	127 (31.9)	220 (36.3)	
	(Missing)	6 (2.9)	4 (1.0)	10 (1.7)	
Center case volume	Up to 25 cases	88 (42.3)	151 (37.9)	239 (39.4)	0.339
	> 25 cases	120 (57.7)	247 (62.1)	367 (60.6)	

*O* open group, *MI* minimally invasive group, *SD* standard deviation, *BMI* body mass index, *ECOG* eastern cooperative oncology group performance status scale, *ACCI* age-adjusted Charlson comorbidity index, *ASA* American society of anesthesiologists physical status classification

^*^Chi-square test for categorical variables, Kruskal–Wallis test for continuous variables (all tested as not normally distributed with Shapiro–Wilk test)

Age, gender distribution, BMI, ACCI score, ECOG status, and center case volume were not significantly different between the two groups. Patients from O group had a higher ASA score as compared with patients from MI group.

The median total anesthetic time was 15.5 min longer in the MI than in the O group and this difference was significant (*p* < 0.004) (Table 2).

**Table 2:** Pathological outcomes

		O	MI	Total	*p* value *
N. cases		208	398	606	
pTNM stage	1	61 (29.3)	139 (34.9)	200 (33.0)	0.198
	2–3	145 (69.7)	256 (64.3)	401 (66.2)	
	(Missing)	2 (1.0)	3 (0.8)	5 (0.8)	
Lymphatic invasion	No	125 (60.1)	285 (71.6)	410 (67.7)	0.650
	Yes	44 (21.2)	89 (22.4)	133 (21.9)	
	(Missing)	39 (18.8)	24 (6.0)	63 (10.4)	
Vascular invasion	No	152 (73.1)	309 (77.6)	461 (76.1)	0.232
	Yes	49 (23.6)	76 (19.1)	125 (20.6)	
	(Missing)	7 (3.4)	13 (3.3)	20 (3.3)	
Length of the specimen (cm)	Median (IQR)	20.0 (15.0–24.6)	19.0 (15.0 to 26.0)	19.0 (15.0 to 25.0)	0.474
Distal margin	Median (IQR)	5.0 (4.0–8.0)	6.0 (4.0 to 8.2)	6.0 (4.0 to 8.0)	0.073
Proximal margin	Median (IQR)	8.0 (6.0–12.0)	8.0 (5.5 to 12.1)	8.0 (5.8 to 12.0)	0.695
Lymph nodes retrieved (*N*)	Median (IQR)	15.0 (12.0–20.0)	15.0 (13.0 to 20.0)	15.0 (12.0 to 20.0)	0.540
Lymph nodes metastatic (*N*)	Median (IQR)	0.0 (0.0–1.0)	0.0 (0.0 to 1.0)	0.0 (0.0 to 1.0)	0.261
Total anesthetic time (minutes)	Median (IQR)	150.0 (115.0–195.0)	170.5 (115.0 to 220.0)	162.0 (115.0 to 210.0)	**0.004**
Splenic resection	No	197 (94.7)	396 (99.5)	593 (97.9)	** < 0.001**
	Yes	11 (5.3)	2 (0.5)	13 (2.1)	

*O* open group, *MI* minimally invasive group, *pT* pathological tumor stage according to 8th TNM system, *pN* pathological nodal stage according to 8th TNM system, *pTNM stage* pathological TNM stage according to 8th TNM system, *R* grade of curative resection, *R0* a microscopically margin-negative resection, in which no gross or microscopic tumor remains in the primary tumor bed, *R1* the removal of all macroscopic disease, but microscopic tumor deposits are present in resection margins or in surgical bed

^*^Chi-square test for all categorical variables with the exception of “splenic resection” for which Fisher’s test has been used, Kruskal–Wallis test for continuous variables (all tested as not normally distributed with Shapiro–Wilk test)

Among these 606 patients, 190? (31.3%) received adjuvant treatment, without significant distribution difference between the 2 groups (*p* value = 0.102).

### Pathological characteristics

Pathological characteristics are reported in Table 2. After removal of pT4 patients, the distribution of pTNM stages (1 vs 2, 3) was comparable in the two arms of patients (*p* = 0.198). The total number of lymph nodes retrieved was similarly high in both groups (median (IQR), 15.0 (12.0 to 20.0) and 15.0 (13.0 to 20.0) in O and MI group respectively). The lymphatic and vascular invasion were comparable (21.2% vs. 22.4%, *p* = 0.650 and 23.6% vs. 19.1%, *p* = 0.232, in O and in MI group, respectively).

Proximal and distal margins’ involvement was not observed in any patients, the median proximal and distal free resection margin length being 8.0 cm and 5.0 cm in O group and 8.0 cm and 6.0 cm in MI group (*p* = 0.695 and *p* = 0.073).

### Recovery and complications

Clavien–Dindo ≥ 3 complications were similar in the two groups (*p* = 1.000) showing a relatively low rate (6.8%) (Table 3). Of the 606 patients with an anastomosis, 22 (3.6%) reported a leak without any differences between the two groups (*p* = 0.664).

**Table 3:** Early postoperative outcomes

		O	MI	Total	*p* value *
No. of cases		208	398	606	
Clavien–Dindo grade > = 3	No	194 (93.3)	370 (93.0)	564 (93.1)	1.000
	Yes	14 (6.7)	27 (6.8)	41 (6.8)	
	(Missing)	0 (0.0)	1 (0.3)	1 (0.2)	
Leak	No	199 (95.7)	385 (96.7)	584 (96.4)	0.664
	Yes	9 (4.3)	13 (3.3)	22 (3.6)	
Medical complication	No	189 (90.9)	372 (93.5)	561 (92.6)	0.319
	Yes	19 (9.1)	26 (6.5)	45 (7.4)	
Type of medical complication	Respiratory	4 (1.9)	3 (0.8)	7 (1.2)	
	Sepsis	1 (0.5)	2 (0.5)	3 (0.5)	
	Cardiac	0 (0.0)	3 (0.8)	3 (0.5)	
	Other types	14 (6.7)	18 (4.5)	32 (5.3)	
Surgical complication	No	184 (88.5)	361 (90.7)	545 (89.9)	0.466
	Yes	24 (11.5)	37 (9.3)	61 (10.1)	
Type of surgical complication	Anastomotic hemorrhage	0 (0.0)	2 (0.5)	2 (0.3)	
	Abdominal collection	1 (0.5)	5 (1.3)	6 (1.0)	
	Bowel Obstruction	2 (1.0)	2 (0.5)	4 (0.7)	
	Pancreatic fistula	0 (0.0)	1 (0.3)	1 (0.2)	
	Significant hemorrhage	2 (1.0)	5 (1.3)	7 (1.2)	
	Surgical site infection	9 (4.3)	6 (1.5)	15 (2.5)	
	Other types	10 (4.8)	16 (4.0)	26 (4.3)	
30-day mortality	No	207 (99.5)	397 (99.7)	604 (99.7)	1.000
	Yes	1 (0.5)	1 (0.3)	2 (0.3)	
Reoperation	No	194 (93.3)	331 (83.2)	525 (86.6)	0.722
	Yes	11 (5.3)	23 (5.8)	34 (5.6)	
	(Missing)	3 (1.4)	44 (11.1)	47 (7.8)	
Length of stay	Median (IQR)	8.0 (7.0–10.0)	7.0 (5.0–9.0)	7.0 (6.0 to 9.0)	** < 0.001**
Conversion	No	208 (100)	357 (89.7)	565 (93.2)	
	Yes	0 (0)	41 (10.3)	41 (6.8)	

*O* open group, *MI* minimally invasive group, *Clavien–Dindo grade ≥ 3* grade ≥ 3 postoperative complications according to Clavien–Dindo Classification, *Conversion* conversion from MI to open surgery

^*^Chi-square test for all categorical variables with the exception of “30-days mortality” for which Fisher’s test has been used, Kruskal–Wallis test for continuous variables (previously checked as not normally distributed with Shapiro–Wilk test)

The conversion rate in MI group was 10.3%.

Postoperative complications, 30-day mortality, and need for reoperation were comparable in the two groups of patients.

Patients treated with an MI approach had a faster perioperative recovery than those submitted to open resection, as reflected by a shorter length of stay (*p* < 0.001) (Table 3).

### Assessing the noninferiority of MI vs. open resection for pathological and clinical outcomes

Successful pathological outcome (a number of LN retrieved ≧12 and both proximal and distal free resection margin lengths ≧5 cm) occurred in 53.2% of O (95% CI 44.6–61.6%) and 58.3% of MI procedures (95% CI 52.9–63.5%) and supported noninferiority (difference 5.1%; 1-sided 95% CI − 4.7% to ∞).

Successful short-term clinical outcome (Clavien–Dindo complications grade < 3 and survival at 30 days after operation) occurred in 93.3% of O (95% CI 89.0–96.3%) and 93.0% of MI procedures (95% CI 90.0–95.3%) and supported noninferiority (difference − 0.3%; 1-sided 95% CI − 4.5% to ∞).

### Survival and recurrence

Survival analyses were computed on 606 patients with information on life status. After a median follow-up time of 4.7 years, 111 patients died, 58 in O (27.9%) and 53 in MI group (13.3%). 58 patients died for other causes than tumor and the cause of death was unknown for 15 patients (8 in O and 7 in MI group). Information on life status was available for all patients.

The 10-year OS was significantly higher in MI than in O group: 76.6% (95% CI 70.1–83.8%) and 62.6% (95% CI 54.0–72.4%), respectively (log-rank test *p* = 0.006) (Fig. 1). The result was not confirmed at Cox regression multivariable analysis (HR 0.72, 95% CI 0.48–1.08, *p* value = 0.109) (Table 4).

**Table 4:** Cox univariable and multivariable analysis of overall survival and cancer-specific mortality

	Cox univariable			Cox multivariable		
	HR	95% CI	*p* value	HR	95% CI	*p* value
Overall survival analysis						
MI (vs. O)	**0.58**	**(0.40–0.84)**	**0.004**	0**.**72	(0**.**48–1**.**08)	0**.**109
Splenic resection	**4.33**	**(2.10–8.92)**	** < 0.001**	**3.28**	**(1.46–7.34)**	**0.004**
ECOG 2 + (vs. 0–1)	**2.82**	**(1.92–4.14)**	** < 0.001**	**1.63**	**(1.06–2.51)**	**0.026**
Males (vs. females)	**2.32**	**(1.52–3.54)**	** < 0.001**	**2.57**	**(1.60–4.12)**	** < 0.001**
Age	**1.09**	**(1.07–1.11)**	** < 0.001**	**1.07**	**(1.05–1.10)**	** < 0.001**
Center caseload > 25 cases	**0.41**	**(0.28–0.61)**	** < 0.001**	**0.45**	**(0.30–0.67)**	** < 0.001**
pTNM 2–3 (vs. 1)	**1.89**	**(1.20–2.97)**	**0.006**	**1.66**	**(1.04–2.65)**	**0.035**
ACCI 6 + (vs. 2–5)	**3.70**	**(2.54–5.39)**	** < 0.001**			
ASA 3 (vs. 1–2)	**1.96**	**(1.34–2.89)**	**0.001**			
Total anesthetic time	**1.00**	**(1.00–1.01)**	**0.050**			
Cancer-specific mortality analysis						
MI (vs. O)	**0.50**	**(0.26–0.96)**	**0.038**	0**.**60		(0**.**29–1**.**23)
Splenic resection	**6.45**	**(2.26–18.38)**	** < 0.001**	**4.26**		**(1.18–15.38)**
ECOG 2 + (vs. 0–1)	1**.**04	(0**.**47–2**.**30)	0**.**919			
Males (vs. females)	**2.06**	**(1.02–4.16)**	**0.044**	**3.20**		**(1.37–7.46)**
Age	**1.04**	**(1.00–1.07)**	**0.033**	1**.**04		(0**.**99–1**.**09)
Center caseload > 25 cases	**0.47**	**(0.25–0.90)**	**0.022**	**0.47**		**(0.23–0.94)**
pTNM 2–3 (vs. 1)	**4.33**	**(1.54–12.21)**	**0.006**	**4.40**		**(1.53–12.62)**
ACCI 6 + (vs. 2–5)	1**.**22	(0**.**59–2**.**53)	0**.**587	0**.**49		(0**.**18–1**.**33)
ASA 3 (vs. 1–2)	1**.**33	(0**.**68–2**.**60)	0**.**401			
Total anesthetic time	1**.**00	(1**.**00–1**.**01)	0.215			

*HR* hazard ratio, *CI* confidence interval, *MI* minimally invasive group, *O* open group, *ECOG* eastern cooperative oncology group performance status scale, *pTNM stage* pathological TNM stage according to 8th TNM system, *ASA* American society of anesthesiologists physical status classification

Deaths for CC were 21 in O (10.1%) and 17 in MI group (4.3%); considering the competing risk of dying for another cause, the proportion of deaths for CC was significantly lower in MI as compared to O group: 7.9% (95% CI 3.4–12.2%) and 16.4% (95% CI: 8.2–23.9%) after 10 years, respectively (Gray’s test *p* = 0.004) (Fig. 1). The result was confirmed at univariable (HR 0.50; 95% CI 0.26–0.96, *p* = 0.038) but not at multivariable Cox regression analysis (HR 0.60, 95% CI 0.29–1.23, *p* value = 0.163) (Table 4).

Twenty-four patients developed tumor recurrence, 10 in O (4.8%) and 14 in MI group (3.5%). Considering the competing risk of dying without recurrence, the difference between the two arms, calculated with Gray Test, was not significant (*p* = 0.434): 5.1% (95% CI: 2.1–8.0%) after 10 years in MI and 6.1% (95% CI 2.0–10.0%) in O group(Fig. 2 suppl).

## Discussion

To date, this report represents the largest series ever published comparing MI versus open segmental colonic resection for SFC aiming to investigate short-term and survival outcomes.

The advantages of MI surgery (MIS) versus open procedures in term of enhanced recovery, hospital stay, postoperative complications, and blood loss have been widely proved for CC in the last 2 decades, becoming a cornerstone for the Enhanced Recovery After Surgery (ERAS) program [26]. Several training programs have been developed to implement MIS spread and to reduce intra- and postoperative complications mostly associated with the learning curve [27–30].

Both in colonic and in rectal cancer treatment programs, despite many RCTs were able to document the superiority of MI vs conventional open approach as concerns short-term outcomes, a real advantage of this procedure with regard to oncological outcomes was never documented, with the exception of a single-center low-volume RCT with several limitations [18, 31–36]. Furthermore, the majority of these RCTs excluded SFC due to its low incidence which makes the related procedures less standardized and often more challenging.

In 2008, Jamali et al. conducted a survey on laparoscopic colorectal surgery to classify the grade of difficulty at each step of the procedure and concluded that the mobilization of SF showed the highest score [37]. A further study from Japan investigated surgery-related factors affecting the difficulty of laparoscopic left colectomy; the authors reported that the mobilization of SF was related with longer surgical time and increased intraoperative complications [38].

In the present study, the overall 30-day mortality was very low (0.3%) without any differences between the two groups, and this should be considered as a proof of the high quality of surgery provided by participating centers. Consistent with data from literature, the MI approach showed a prolonged operation time as a consequence of the more demanding procedure; however, a substantially quite low conversion rate was reported.

Many authors have reported that complications of MI and open left colectomies were comparable, because the critical steps of the two interventions are essentially the same with a different approach [31, 35, 36]. Moreover, MIS has been reported in other studies to harbor a significant reduction not only of medical but even of severe surgical complications, leading to a relevant improvement of patients’ recovery as compared to conventional treatment [39]. Consistent with these data, in our study, there was no difference in the anastomotic leakage or in the other surgical complications between the two groups, and reoperation rate was also comparable. Furthermore, in our study, the MI approach met the criterion for noninferiority for short-term clinical outcome (Clavien–Dindo grade < 3 postoperative complications and postoperative mortality) compared with open resection.

MIS has often been investigated with the aim of verifying whether oncological outcomes could be reproduced as provided by standard open procedures. Indeed, oncological quality of surgery performed on patients affected by colorectal cancer has always been considered as a primary endpoint in every clinical trial. Effectively, in the present study, the main operative factors that globally characterize the high quality of surgery, including the number of lymph nodes harvested and the length of margins of the resected bowel, in both groups equaled or even exceeded those reported in several other studies, without any differences between O and MI arms [40]. Additionally, this study could show that the MI approach met the criterion for noninferiority for pathological outcomes (number of LNs retrieved at least 12, proximal and distal free resection margin length of at least 5 cm) compared with open resection.

Overall survival outcomes observed in our study and reported in our previous paper are better as compared to literature data, where SFCs are usually described with the lowest results among all colorectal tumor locations [3, 41]. Nevertheless, the survival advantage observed after univariable analysis in MI arm could not be confirmed by further multivariable analysis, and definitive findings from the present study could not demonstrate that MI resection harbors an independent protective effect on the oncological outcomes in terms of OS, disease specific mortality, and recurrence, which were found comparable. Nonetheless, taking into consideration all its limitations and biases, to our best knowledge, this is the first study, suggesting that patients’ survival could be improved after MI colectomy over open resection for nonmetastatic SFC; there is some evidence supporting the oncological benefits of MI approach, including reduction of surgical trauma and cancer manipulation, leading to better preservation of the early postoperative cellular immune action and reduced release of inflammatory factors such as cytokines; less postoperative complications leading to enhanced recovery and less blood loss with reduced need for transfusions [42, 43]. Some of these evidences have been proved in preclinical and clinical trials [44–46].

This study has several limitations. First, it is a retrospective report with its inherent bias. Second, it included patients through a long time period characterized by several improvements in many fields including endoscopic preoperative treatment [47], surgical and technological innovations, intraoperative lymphatic and vascular scintigraphic mapping [48], pathological analysis (molecular classification) [49, 50], postoperative management (ERAS) [26], and above all novel adjuvant treatments [51, 52]. Nonetheless, the study involved the largest cohort of patients with SFC ever reported in the literature to date and included 30 referral hospitals for colorectal cancer. The quality of surgery performed resulted in 5-year OS rates of 79.2%, which is favorable as compared to those usually reported thus far.

In conclusion, among patients with cancer of the splenic flexure, both the open and the MI procedure were found to have comparable results of OS, cancer-specific mortality, and recurrence. Furthermore, the use of laparoscopic resection compared with open resection succeeded to meet the noninferiority criterion both for short-term clinical and pathological outcomes. Data provided by this study are in line with the spread of MIS for colorectal cancer through teaching and referral centers. In addition, they represent an updated benchmark for MI surgery as provided in tertiary care facilities.

## Supplementary Information

Below is the link to the electronic supplementary material.

Supplementary file1 Fig 1 suppl. Study Flowchart. After exclusion of patients with ASA IV grade, pT4 disease and those submitted to emergency surgery, a total of 606 patients were definitely included in the study. (JPG 193 KB)

Supplementary file2 Figure 2 suppl. Recurrence by type of surgical approach. After 10 years from resection tumor recurrence rate was 5.1% (95% CI: 2.1%-8.0%)in MI group and 6.1% (95% CI: 2.0%-10.0%) in O group, and the difference between the two arms was not significant (Gray’s test p=0.434). (JPG 95 KB)

Supplementary file3 (DOCX 17 KB)
