# Correction: Minimally invasive vs. open segmental resection of the splenic flexure for cancer: a nationwide study of the Italian Society of Surgical Oncology-Colorectal Cancer Network (SICO-CNN)

**DOI:** 10.1007/s00464-023-10102-0

**Published:** 2023-05-09

**Authors:** Maurizio Degiuli, Monica Ortenzi, Mariano Tomatis, Lucia Puca, Desiree Cianflocca, Daniela Rega, Annalisa Maroli, Ugo Elmore, Francesca Pecchini, Marco Milone, Roberta La Mendola, Erica Soligo, Simona Deidda, Domenico Spoletini, Diletta Cassini, Alessandra Aprile, Michela Mineccia, Herald Nikaj, Francesco Marchegiani, Fabio Maiello, Cristina Bombardini, Michele Zuolo, Michele Carlucci, Luca Ferraro, Armando Falato, Alberto Biondi, Roberto Persiani, Patrizia Marsanich, Daniele Fusario, Leonardo Solaini, Sara Pollesel, Gianluca Rizzo, Claudio Coco, Alberto Di Leo, Davide Cavaliere, Franco Roviello, Andrea Muratore, Domenico D’Ugo, Francesco Bianco, Paolo Pietro Bianchi, Paola De Nardi, Marco Rigamonti, Gabriele Anania, Claudio Belluco, Roberto Polastri, Salvatore Pucciarelli, Sergio Gentilli, Alessandro Ferrero, Stefano Scabini, Gianandrea Baldazzi, Massimo Carlini, Angelo Restivo, Silvio Testa, Dario Parini, Giovanni Domenico De Palma, Micaela Piccoli, Riccardo Rosati, Antonino Spinelli, Paolo Delrio, Felice Borghi, Marco Guerrieri, Rossella Reddavid

**Affiliations:** 1 University of Turin, Department of Oncology, San Luigi University Hospital, Div of Surgical Oncology, Orbassano, Turin, Italy; 2 Clinica Chirurgica Universita’ Politecnica delle Marche, Ospedali Riuniti, Ancona, Italy; 3 BSIT, Department of Oncology, University of Turin, Orbassano, Turin, Italy; 4 Department of Surgery, S. Croce e Carle Hospital, Cuneo, Italy; 5 Department of General and Emergency Surgery, Azienda Ospedaliero Universitaria, Città della Salute e della Scienza, Turin, Italy; 6 Colorectal Surgical Oncology, Abdominal Oncology Department, Fondazione Giovanni Pascale IRCCS, Naples, Italy; 7 Colon and Rectal Surgery Division, Humanitas Clinical and Research Center, Via Alessandro Manzoni, 56, Rozzano, 20089 Milan, Italy; 8 Division of Gastrointestinal Surgery, Vita Salute University, San Raffaele Hospital, 20132 Milan, Italy; 9 Unita’ Operativa di chirurgia generale, d’urgenza e nuove tecnologie, OCSAE, Azienda Ospedaliero Universitaria di Modena, Modena, Italy; 10 Department of Clinical Medicine and Surgery, Department of Gastroenterology, Endocrinology and Endoscopic Surgery, University of Naples “Federico II”, Naples, Italy; 11 General Surgery Unit, Santa Maria della Misericordia Hospital, Rovigo, Italy; 12 S.C. Chirurgia Generale, Ospedale S. Andrea, Vercelli, Italy; 13 Chirurgia Coloproctologica-AOU Cagliari, Dipartimento di Scienze Chirurgiche, Università di Cagliari, Cagliari, Italy; 14 UOC Chirurgia Generale, Ospedale S. Eugenio, Piazzale dell’Umanesimo, 10, 00144 Rome, Italy; 15 Unità Operativa Complessa di Chirurgia Generale, P.O. SSG, ASST NORD MILANO, Milan, Italy; 16 Surgical Oncology Surgery, IRCCS Policlinico San Martino, Genoa, Italy; 17 Department of General and Oncological Surgery, ”Umberto I” Mauriziano Hospital, Turin, Italy; 18 SCDU Clinica Chirurgica, General Surgery Department, AOU “Maggiore Della Carità” Hospital, Novara, Italy; 19 Department of Surgical, Oncological, and Gastroenterological Sciences, University of Padua, Padua, Italy; 20 Department of Surgery, General Surgery Unit, Hospital of Biella, Biella, Italy; 21 Department of Surgical Morphology and Experimental Medicine, AOU Ferrara, Ferrara, Italy; 22 General Surgery Division, “Valli del Noce” Hospital, Cles, Provincial Agency for Health Services (APSS), Trento, Italy; 23 Gastrointestinal Surgery, San Raffaele Hospital, 20132 Milan, Italy; 24 Division of General and Robotic Surgery, Dipartimento di Scienze della Salute, Università di Milano, 20142 Milan, Italy; 25 General Surgery Unit, San Leonardo Hospital, ASL-NA3sud, Castellammare di Stabbia, Naples, Italy; 26 Fondazione Policlinico Gemelli, IRCCS, AREA di Chirurgia Addominale, Rome, Italy; 27 Surgical Department, Edoardo Agnelli Hospital, Pinerolo, Italy; 28 UOC General and Oncological Surgery, University of Siena, Siena, Italy; 29 General and Oncologic Surgery, Morgagni-Pierantoni Hospital, Ausl Romagna, Forlì, Italy; 30 Fondazione Policlinico Universitario A. Gemelli, IRCCS, Chirurgia Generale Presidio Columbus, Rome, Italy; 31 UOC di Chirurgia, Ospedale “San Camillo”, Trento, Italy; 32 Department of Surgery, Misericordia Hospital, Grosseto, Italy; 33 Department of Surgical Oncology, CRO Aviano, National Cancer Institute, IRCCS, Aviano, Italy; 34 UOC Chirurgia Generale, Ospedale S. Eugenio, Piazzale dell’umanesimo, 10, 00144 Rome, Italy; 35 IRCCS Humanitas Research Hospital, Via Manzoni 56, 20089 Rozzano, Milan Italy; 36 Department of Biomedical Sciences, Humanitas University, Via Rita Levi Montalcini 4, 20072 Pieve Emanuele, Milan Italy; 37 Oncological Surgery, Candiolo Cancer Institute-FPO-IRCCS, Candiolo, 10060 Torino, Italy; 38 Department of Oncology, Head Surgical Oncology and Digestive Surgery, University of Torino, San Luigi University Hospital, Regione Gonzole 10 Orbassano, 10043 Turin, Italy

**Correction to: Surgical Endoscopy (2022) 37:977-988** 10.1007/s00464-022-09547-6

The original online version of this article was revised to correct the affiliations of author Antonino Spinelli. The correct affiliations are Department of Biomedical Sciences, Humanitas University, Via Rita Levi Montalcini 4, 20072 Pieve Emanuele, Milan, Italy; IRCCS Humanitas Research Hospital, via Manzoni 56, 20089 Rozzano, Milan, Italy.

The original article has been corrected.

