# Supplementary figures and images for: Minimally invasive vs. open segmental resection of the splenic flexure for cancer: a nationwide study of the Italian Society of Surgical Oncology-Colorectal Cancer Network (SICO-CNN)
Source: Surg Endosc. 2022 Sep 9;37(2):977–88. doi: 10.1007/s00464-022-09547-6 (PMC9944710; doi:10.1007/s00464-022-09547-6)

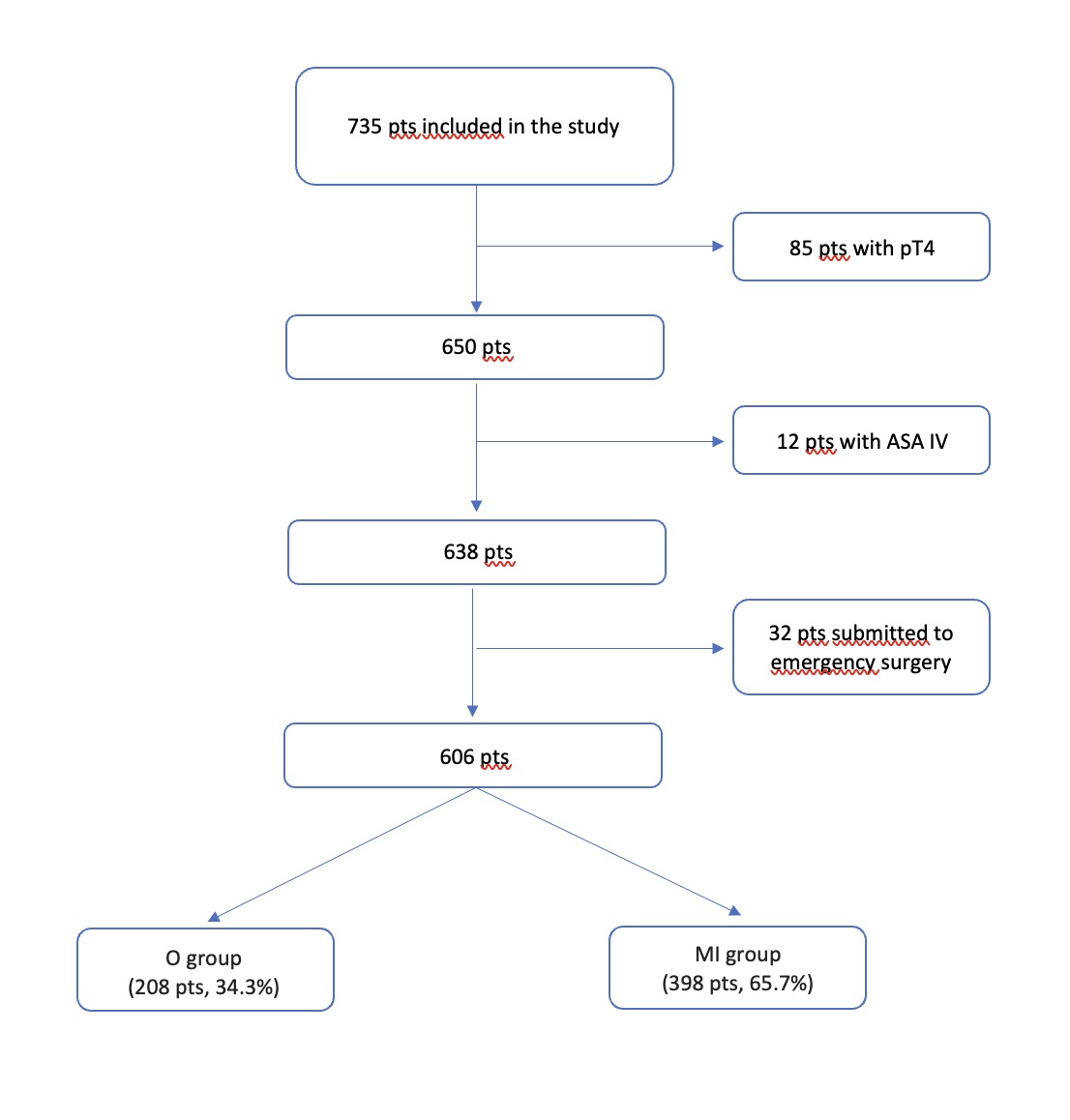

Supplement: Supplementary file 1 — Supplementary file1 Fig 1 suppl. Study Flowchart. After exclusion of patients with ASA IV grade, pT4 disease and those submitted to emergency surgery, a total of 606 patients were definitely included in the study. (JPG 193 KB) [file 464_2022_9547_MOESM1_ESM.jpg]

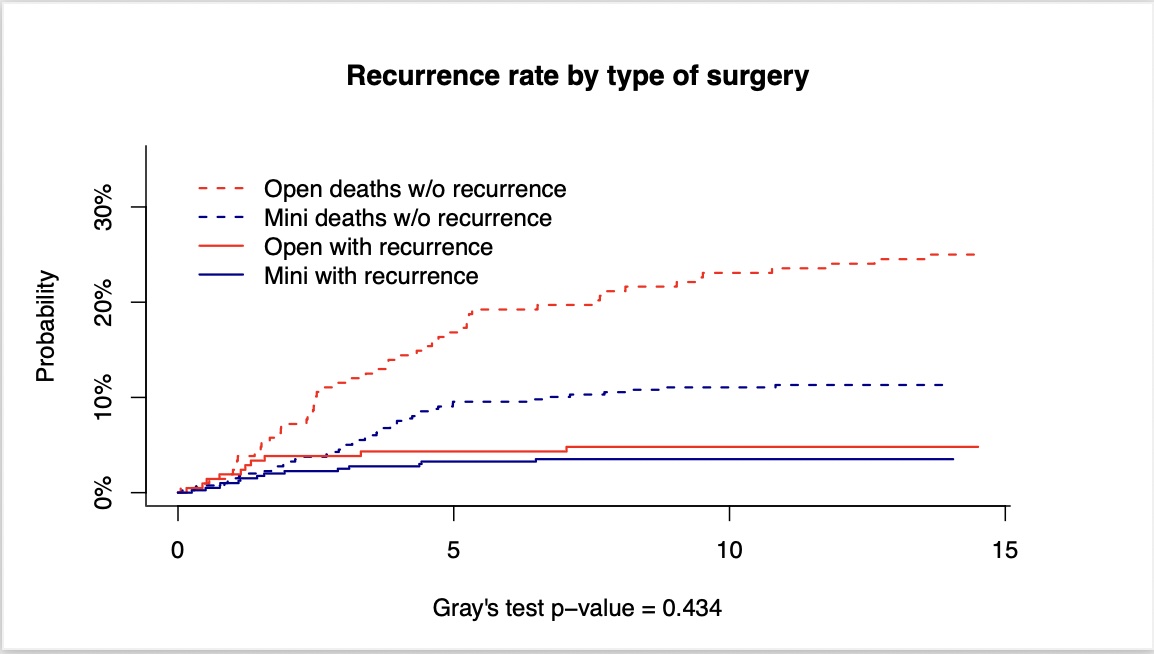

Supplement: Supplementary file 2 — Supplementary file2 Figure 2 suppl. Recurrence by type of surgical approach. After 10 years from resection tumor recurrence rate was 5.1% (95% CI: 2.1%-8.0%)in MI group and 6.1% (95% CI: 2.0%-10.0%) in O group, and the difference between the two arms was not significant (Gray’s test p=0.434). (JPG 95 KB) [file 464_2022_9547_MOESM2_ESM.jpg]
