# Supplementary material for: Minimally invasive vs. open segmental resection of the splenic flexure for cancer: a nationwide study of the Italian Society of Surgical Oncology-Colorectal Cancer Network (SICO-CNN)
Source: Surg Endosc. 2022 Sep 9;37(2):977–88. doi: 10.1007/s00464-022-09547-6 (PMC9944710; doi:10.1007/s00464-022-09547-6)
Supplement: Supplementary file 3 — Supplementary file3 (DOCX 17 KB) [file 464_2022_9547_MOESM3_ESM.docx]

**Table 1 suppl.** Details of participating centers collecting data.

| **Center** | **Local P.I.** | **pts collected** |
| --- | --- | --- |
| Ancona - Ospedali riuniti | M. Guerrieri | 107 |
| Napoli - IRCCS | P. Delrio | 43 |
| Cuneo - Santa Croce | F. Borghi | 41 |
| Milano - Humanitas | A. Spinelli | 38 |
| Napoli - Federico II | G. D. De Palma | 32 |
| Modena OCSAE | M. Piccoli | 27 |
| Vercelli - Ospedale sant'Andrea | S. Testa | 26 |
| Milano - HSR | R. Rosati | 24 |
| Cagliari - AOU | L. Zorcolo | 23 |
| Rovigo - Osp S. Maria Misericordia | Parini | 21 |
| Abano Terme | Baldazzi | 20 |
| Orbassano - San Luigi | M. Degiuli | 20 |
| Torino - Mauriziano | A Ferrero | 19 |
| Novara - AOU Maggiore della Carità | S. Gentilli | 17 |
| Padova - AOU | S. Pucciarelli | 16 |
| Aviano - CRO | G. Bertola | 15 |
| Genova – Osp. S. Martino | S. Scabini | 15 |
| Grosseto | P. P. Bianchi | 13 |
| Biella | R. Polastri | 12 |
| Ferrara | G. Anania | 12 |
| Napoli - IRCCS 2 | Bianco | 12 |
| Milano – HSR 2 | P. De Nardi | 10 |
| Trento - Ospedale Civile di Cles | M. Rigamonti | 9 |
| Roma - Policlinico Gemelli | D. D’Ugo | 9 |
| Roma - S. Eugenio | Sica | 9 |
| Forlì | Cavaliere | 4 |
| Siena | F. Roviello | 4 |
| Verona - AOU | A Di Leo | 4 |
| Pinerolo - Ospedale Agnelli | A Muratore | 2 |
| Roma - Cattolica | C. Coco | 2 |
